# Understanding the impact of the COVID-19 pandemic on refugee communities in San Diego, California: A participatory action research cross-sectional study

**DOI:** 10.1016/j.ssmph.2022.101110

**Published:** 2022-04-30

**Authors:** Lotus McDougal, Jeanine Erikat, Homayra Yusufi, Ramla Sahid, Samantha Streuli, Rebecca Fielding-Miller

**Affiliations:** a Division of Infectious Diseases and Global Public Health, School of Medicine, University of California, San Diego, USA; b Partnership for the Advancement of New Americans, San Diego, USA; c Herbert Wertheim School of Public Health and Human Longevity Science, University of California, San Diego, USA

**Keywords:** COVID-19, Refugees, Job loss, Mental health, San Diego, California

## Abstract

**Background:**

While the COVID-19 pandemic has impacted people worldwide, refugee communities are particularly vulnerable to the pandemic's social, economic and health impacts. This study assessed factors associated with increases in adverse community effects of COVID-19 in a refugee community in California.

**Methods:**

This study uses data from a cross-sectional survey developed and administered as part of a participatory action research project by a refugee community organization in San Diego, California. Data was collected between September and November 2020 in a sample of refugee community members (n = 517). Multivariable Poisson regression models measured associations between sociodemographic and acculturation measures with seven adverse community effects overall and stratified by duration of residence in the United States. Adverse community effects included job/wage loss, bank/cash access barriers, food insecurity, school interruptions, household violence, substance misuse and poor mental health.

**Results:**

Refugee community members reported an average of 2.1 adverse community effects that worsened during the COVID-19 pandemic, with job/wage loss and poor mental health the most prevalent (84% and 49%). Characteristics associated with reporting increased numbers of adverse community effects included being younger, female, childless, not actively seeking employment, living in the US for six or more years and speaking English at home. Stratified analyses show that these associations were concentrated in refugees who had lived in the US for at least six years.

**Conclusion:**

Refugee communities have experienced pervasive job losses and worsening mental health during the COVID-19 pandemic, and these effects are concentrated in respondents who have lived in the US for six or more years. Additional targeted support is needed to ensure that refugees who have lived in the US for longer durations have the financial and social support needed to cope with the unprecedented challenges brought on by the COVID-19 pandemic.

## Highlights

• Refugee community job/wage loss and mental health worsened during the pandemic.
• Higher COVID-19 job/wage loss in San Diego refugee community than most Californians.
• Adverse community effects concentrated in more long-term immigrants.
• Upstream, structural shifts needed to prevent downstream adverse effects.

## 1 Introduction

The COVID-19 pandemic has created severe disruptions across the globe, and will continue to do so for many years. While individuals who contract COVID-19 are most directly affected, whole communities have been broadly impacted by job and wage losses, supply chain shortages, increased food costs and service limitations (Han & Hart, 2021; Wolfson & Leung, 2020b). Not surprisingly, communities that have been made socially vulnerable by historical experiences of racism and underinvestment have experienced the highest rates of morbidity, mortality, and community-level impact. In the United States, in age-adjusted analyses, Black Americans are 3.5 times more likely to be hospitalized and 2.4 times more likely to die than their White counterparts and Hispanic Americans are 2.8 and 2.0 times more likely to be hospitalized or die, respectively (Kaiser Family Foundation, 2021). Low-income racialized minority communities have experienced higher rates of food insecurity, energy insecurity, learning loss, and trauma (Goudeau et al., 2021; Lund, 2021; Marron, 2021; Memmott et al., 2021; Wolfson & Leung, 2020a).

The social and structural determinants of health that influence health outcomes and experiences for minorities are further amplified in refugee communities (Feinberg et al., 2021; Krieger, 2014). Refugee communities in the United States sit at the intersection of multiple structural drivers of COVID-19 harm. Levels of acculturation, or the degree to which an individual incorporates elements of a new culture, vary substantially within refugee communities based on multiple factors, including their duration of residence in the United States, and their social connectedness with individuals outside of their culture of origin (Guler & Berman, 2019; Padilla, 1980). These refugee communities are made up of individuals with diverse immigration experiences and cultural backgrounds that influence mental health and racialized vulnerabilities (Pogarell et al., 2019; Shi et al., 2021). They are also dynamic, not only because new members may join, but because community members change as they spend longer periods of time in host countries, acculturation progresses, and younger generations are born that more fluidly straddle different cultures (Abu-Kaf et al., 2021; Fazel et al., 2012; Lazarevic et al., 2012).

In addition to the dynamic structure of refugee communities themselves, available support mechanisms vary by location, duration of residence, community and organizational resources (Brown& Scribner, 2014; Lacroix et al., 2015; Zetter & Pearl, 2000). The complexity of navigating these support pathways for refugees and their families is substantial, particularly in settings that are unfamiliar and organizational support systems that vary from one community to the next (Brown& Scribner, 2014). These complexities are to a large extent mirrored in barriers faced by refugee community organizations striving to provide the highest feasible level of support, who must function within highly administrative and fluctuating resource and policy pathways (Zetter & Pearl, 2000). These challenges underscore the need to promote upstream structural changes to improve health outcomes in a participatory framework. This participatory approach, particularly with refugee communities, has been inconsistently applied to date (Filler et al., 2021).

The experiences of refugees who have resettled into communities in the United States are increasingly important to understand in the context of the government's current commitment to expand the resettlement program. As of January 2022, more than 76,000 Afghan refugees have been, or are in the process of being, resettled by the Biden Admnistration, with the goal of admitting 125,000 more refugees during fiscal year 2022 (Blinken, 2021; The White House, 2022). This commendable initiative marks a more than eight-fold increase over resettlement numbers under the prior Administration (US Department of State, 2021), an expansion that should be undertaken with deeper understanding of the complex challenges manifested in this vulnerable community due the ongoing COVID-19 pandemic.

San Diego is an important setting in which to understand the ways in which the COVID-19 pandemic has affected refugee communities specifically. In 2020–21, California accepted more refugees than any other state in the country (Refugee Processing Center, 2021). San Diego county resettles the most refugees in the state of California, accepting more than 1900 refugees in fiscal year 2020-21 alone (County of San Diego, 2021). The Partnership for the Advancement of New Americans (PANA) is a refugee-led research, public policy, and community organizing hub in San Diego, California that works to serve this high volume of resettled refugees through a broad range of services and activities, including biennial community needs surveys. The objective of this study is to understand the ways that the COVID-19 pandemic has adversely affected refugee communities served by PANA in San Diego, and whether those effects differ based on duration of time in the US.

## 2 Methods

### 2.1 Survey design and data collection

Data collection was initiated by PANA as part of their biannual survey of refugee experiences in San Diego county. The research and survey design process was designed in line with a community based participatory research (CBPR) framework, particularly the intervention and research process and outputs described by Wallerstein et al. (Belone et al., 2016; Cashman et al., 2008; Sanchez et al., 2021; Wallerstein & Duran, 2010). Individual items were selected by centering community knowledge and preferences and then selecting measures that would increase the likelihood of generalizability and comparability between the refugee community and broader US population (e.g., selecting items from the US Census, American Community Survey, and the National Institute of Health PhenX toolkit) (Hamilton et al., 2011). The full survey was iteratively piloted with community member researchers and modified in discussion with PANA staff until study leadership agreed that each survey item was both useful and culturally appropriate, and that the overall length and structure would be acceptable to community participants. These conversations included translational considerations and issues of cultural meaning with individual survey items (i.e., questions related to mental health, violence, and belonging).

The survey was administered between September and November 2020, and contained questions pertaining to a range of topics relevant to refugee communities, including health, employment, education and COVID-19. Trained survey interviewers, who were themselves members of the refugee community, administered the survey to study participants in the participants’ language of choice (English, Arabic, Burmese, Dari, Karen, Karenni, Oromo, Pashto, Somali or Swahili). The survey was administered primarily over the phone with several in-person data collection events in open public spaces such as public parks. Interviewers entered data directly into Qualtrics, an online computer-assisted survey platform. The PANA-designed survey was complemented by community meetings and online conversations to ensure refugee community members had opportunities to provide their perspectives and feedback on a range of topics including COVID-19, housing, employment, education, safety and belonging. These conversations also enabled community members and PANA to discuss action-oriented goals to address concerns brought up during these meetings.

Survey respondents were members of the refugee community in San Diego who had engaged with PANA in the previous year and provided their contact information. In total, 517 respondents ages 18–96 were interviewed and eligible for inclusion in this analysis.

### 2.2 Measures

Our outcome of interest was adverse community effects of COVID-19. This was derived from the survey question *“In your community, which of the following has increased since the start of the COVID-19 pandemic and the social containment efforts to control the spread of the virus (e.g., lockdown, curfews, etc.)?”* This question had multiple response options, which included “*Many people have lost their jobs or their ability to earn wages, and some have lost their business”, “People are unable to bank or get cash for daily expenses”, “Families cannot afford to buy enough food to eat”, “Many schools have closed, and the children are unable to continue their studies at home”, “There is more violence in the household”, “People are drinking more and/or using drugs more”* and “*More people are becoming very anxious or depressed”*. Respondents were able to select as many of these response options as they felt were applicable. The primary outcome of interest was an aggregate index of all of these seven possible responses, ranging from 0 to 7, depending on how many areas people noted increases in since the start of the pandemic. The secondary outcomes of interest were the individual areas in which communities may have experienced the effects of COVID-19.

Independent variables included sociodemographic and acculturation measures. Assessed sociodemographics were respondent age, gender, number of children (none/1–2/3 or more) and past week employment. Past week employment measured whether or not respondents had worked for pay in the prior seven days, and if they had not, whether they were looking for work or not. In line with prior research, proxy measures of acculturation were number of years in the US (5 or fewer/6 or more) and whether or not the respondent spoke English in their home (Alidu & Grunfeld, 2018; Anderson et al., 2016; Cruz et al., 2008).

An additional measure that is presented descriptively but not included in multivariable analyses due to the conceptual challenge with selecting a referent group is respondent's country of birth. This was asked as an open-ended question during survey administration.

### 2.3 Analysis

Recognizing the democratic and reflective principles underlying participatory research, all analytic decisions were made through iterative, collaborative discussions between PANA and the UCSD team (Bergold & Thomas, 2012; Rix et al., 2021). These conversations were intended to ensure that the primary endpoints and analytic approach was consistent with PANA's institutional mission to “fight to enhance the full economic, social, and civic inclusion of refugees in the San Diego region, throughout Caliornia, and across the country” (Partnership for the Advancement of New Americans, 2022).

We conducted descriptive analyses to assess the overall prevalence of adverse community effects of COVID-19, as well as the prevalence of each individual effect (e.g. job/wage loss, bank/cash barriers, etc.). Prevalence estimates are presented overall, as well as for each of the independent variables.

As the number of adverse community effects was a count outcome, multivariable Poisson regressions were used to assess the relationship between independent variables and the number of adverse community effects reported. These Poisson models are also presented stratified by respondent's duration of residence in the United States to assess the influence of time since immigration on these relationships, given the importance of this factor in influencing refugee experiences (Hasanović et al., 2020; Sheikh & Anderson, 2018). Multivariable logistic regressions were used to assess the relationship between independent variables and each of the binary secondary outcomes of interest, both overall and stratified by duration of residence in the United States. Finally, a post-hoc analysis stratified the overall multivariable Poisson model by country of birth for the three largest groups in these data (respondents born in Syria, Somalia and Afghanistan). No collinearity existed between variables using a cutoff of VIF = 5. The maximum VIF was 1.7, with a mean of 1.4.

Four of the seven assessed secondary outcomes (job/wage loss, food insecurity, substance misuse, and poor mental health) were also included in a survey administered in March 2021 to the California population at large, the Cal-VEX survey (NORC at the University of Chicago; Center on Gender Equity and Health, 2021). The prevalence of these four outcomes was thus descriptively compared with estimates for California as a whole, and the Southern California region specifically, as the Cal-VEX survey was designed to produce representative estimates of both geographies. The University of California San Diego Center on Gender Equity and Health was involved in the collection of the Cal-VEX survey data, and provided the southern California regional estimates used in this manuscript, in addition to the previously disseminated California statewide data (NORC at the University of Chicago; Center on Gender Equity and Health, 2021).

### 2.4 Ethics

These data were collected as part of the PANA biannual Refugee Experiences Report. The study was reviewed and approved by the University of California, San Diego Institutional Review Board. All elements of study and survey design were led by PANA staff (RS, JE, HY) with technical support from RFM. Participants provided informed consent before completing the survey and at completion were given a $15 gift card to thank them for their time and expertise.

## 3 Results

### 3.1 Descriptive results

#### 3.1.1 San Diego refugee community

Nearly one-third of refugee community respondents (29.8%) were born in Syria (Table 1). These Syrian refugees arrived in the US an average of 4 years ago and the majority identified as male (85%). Just over one-fifth of respondents (23%) were born in Somalia; Somali refugee respondents arrived in the US an average of 19 years ago and the majority identified as female (75%). Respondents born in Afghanistan (17% of the sample) arrived in the US an average of 5 years ago and the majority identified as male (90%). Respondents born in Myanmar (8% of the sample) arrived in the US an average of 8 years ago and the majority identified as female (78%). Overall, respondents were on average 40 years old, and were majority (51%) unemployed and not looking for work. Sixty-eight percent of respondents had at least one child under the age of 18 for whom that they were responsible, with an average of 6 children (among those with children). Just over half of all respondents (51%) were relatively new immigrants, having been in the US for five years or fewer, and two-thirds (67%) did not speak English at home.

**Table 1:** Descriptive sample characteristics.

	Total		Overall number of adverse community effects		Individual adverse community effects													
					Job/Wage loss		Bank/cash barriers		Food insecurity		School interruptions		Household violence		Substance misuse		Poor mental health	
	N	%	Mean	SD	N	%	N	%	N	%	N	%	N	%	N	%	N	%
*CA State*	2183		-	-	1228	56.6			781	34.7					730	35.8	1080	52.3
*Southern CA region*	1143		-	-	625	54.8			365	31.8					388	36.9	552	51.8
Total	517	100	2.1	1.7	433	83.8	76	14.7	94	18.2	115	22.2	40	7.7	57	11.0	253	48.9
**Sociodemographics**																		
Country of birth																		
Syria	153	29.8	1.7	0.8	140	91.5	9	5.9	1	0.7	15	9.8	1	0.7	10	6.5	79	51.6
Somalia	116	22.6	1.9	2.1	84	72.4	18	15.5	29	25.0	20	17.2	14	12.1	14	12.1	42	36.2
Afghanistan	86	16.7	1.2	0.7	78	90.7	1	1.2	2	2.3	5	5.8	2	2.3	1	1.2	18	20.9
Myanmar	41	8.0	3.0	1.3	38	92.7	8	19.5	8	19.5	22	53.7	3	7.3	7	17.1	35	85.4
Iraq	21	4.1	1.9	1.0	18	85.7	1	4.8	1	4.8	5	23.8	1	4.8	1	4.8	13	61.9
Ethiopia	17	3.3	2.5	1.7	13	76.5	3	17.7	8	47.1	4	23.5	1	5.9	1	5.9	12	70.6
USA	31	6.0	4.1	2.7	23	74.2	17	54.8	22	71.0	17	54.8	11	35.5	14	45.2	22	71.0
Other^a^	49	9.5	3.1	2.0	38	77.6	18	36.7	23	46.9	27	55.1	7	14.3	9	18.4	30	61.2
Age (years) b	40.2	13.0	-	-	39.9	12.8	33.9	12.1	34.4	15.5	35.3	12.6	32.0	10.9	33.2	11.6	39.0	12.8
Gender																		
Male	285	55.7	1.8	1.3	260	91.2	26	9.1	27	9.5	33	11.6	11	3.9	21	7.4	123	43.2
Female	227	44.3	2.5	2.0	171	75.8	49	21.6	67	29.5	82	36.1	29	12.8	36	15.9	129	56.8
Children																		
None	162	32.3	2.6	2.1	133	82.1	39	24.1	55	34.0	49	30.3	25	15.4	32	19.8	92	56.8
1-2	126	24.7	2.2	1.8	111	88.1	19	15.1	22	17.5	30	23.8	11	8.7	16	12.7	67	53.2
3 or more	222	43.5	1.6	1.0	188	84.7	18	8.1	17	7.7	35	15.8	4	1.8	9	4.1	94	42.3
Past week employment																		
Yes	134	26.4	2.1	1.7	122	91.0	23	17.2	21	15.7	36	26.9	8	6.0	15	11.2	60	44.8
None, looking for work	114	22.5	1.9	1.6	91	79.8	11	9.7	28	24.6	16	14.0	11	9.7	9	7.9	45	39.5
None, not looking for work	259	51.1	2.2	1.7	216	83.4	42	16.2	45	17.4	62	23.0	21	8.1	33	12.7	144	55.6
**Acculturation**																		
Years in the US																		
5 or fewer	258	51.1	1.6	1.0	228	88.4	18	7.0	15	5.8	35	13.6	4	1.6	12	4.7	113	43.8
6 or more	247	48.9	2.5	2.1	196	79.4	54	21.9	75	30.4	77	31.2	34	13.8	42	17.0	134	54.3
English at home																		
No	345	66.7	1.7	1.2	289	83.8	31	9.0	36	10.4	51	14.8	6	1.7	19	5.5	166	48.1
Yes	172	33.3	2.7	2.3	144	83.7	45	26.2	58	33.7	64	37.2	34	19.8	38	22.1	87	50.6

a Democratic Republic of the Congo, Kenya, Jordan, Algeria, Burundi, Egypt, Lebanon, Malaysia, Morocco, Pakistan, Sudan, South Sudan, Thailand, Yemen, Zimbabwe.

b Mean, standard deviation.

On average, respondents indicated that 2.1 (of 7) assessed areas worsened in their communities during the COVID-19 pandemic; increases were highest in job/wage loss (83.8%) and poor mental health (48.9%), and lowest in household violence (7.7%) and substance misuse (11.0%) (Table 1).

#### 3.1.2 Comparison with California

Levels of worsening community mental health in this refugee community respondent sample were comparable to those from a broader sample representing southern California regionally, and California statewide (51.8% and 52.3%, respectively) (Table 2). Community reports of job or wage loss were lower in southern California and California statewide (54.8% and 56.6%, respectively), than that seen in refugee community respondents in San Diego. San Diego refugee community respondents who had been in the US for six or more years had comparable levels of food insecurity (30.4%) to that seen in the southern California region (31.8%) and state (34.7%), while newer immigrants responding to this survey noted substantially lower levels of food insecurity in their communities (5.8%). Increases in community substance misuse noted by refugee respondents was lower than that seen in the region or state, irrespective of time in the US.

**Table 2:** Descriptive comparison of refugee respondents in San Diego with representative samples of California and the Southern California region.

	Total	Job/Wage loss		Food insecurity		Substance misuse		Poor mental health	
	N	N	%	N	%	N	%	N	%
California statewide^a^	2183	1228	56.6	781	34.7	730	35.8	1080	52.3
Southern California region^a^	1143	625	54.8	365	31.8	388	36.9	552	51.8
San Diego refugee community (total sample)	517	433	83.8	94	18.2	57	11.0	253	48.9
In US for ≤5 years	258	228	88.4	15	5.8	12	4.7	113	43.8
In US for ≥6 years	247	196	79.4	75	30.4	42	17.0	134	54.3

a Data from Cal-VEX survey survey (NORC at the University of Chicago; Center on Gender Equity and Health, 2021).

### 3.2 Multivariable results

#### 3.2.1 Number of adverse events

Multivariable results for our primary outcome of interest, number of areas in which respondents noted adverse community effects, indicated that for every year of age, the percent change in the incidence rate of adverse effects in the community decreased by 1.3% (p < 0.001) (Table 3). Females were more likely to report increases in overall rates of adverse community effects than men (aIRR = 1.17, p < 0.01), and respondents with three or more children reported decreases in the overall rates of adverse community effects relative to those with no children (aIRR = 0.83, p < 0.05). Relative to people who were unemployed but looking for work, those who were employed or unemployed and not looking for work reported increases in the incidence rate of adverse community effects (aIRR = 1.34, p < 0.01 and aIRR = 1.49, p < 0.001, respectively).

**Table 3:** Multivariable Poisson regression models exploring factors associated with the number of adverse community effects experienced during the COVID-19 pandemic by refugee communities in San Diego, California.

	Total		In US for 5 or fewer years		In US for 6 or more years	
	aIRR	SE	aIRR	SE	aIRR	SE
**Sociodemographics**						
Age (years)	0.987***	(0.00263)	0.995	(0.00388)	0.985***	(0.00344)
Gender						
Male	REF	REF	REF	REF	REF	REF
Female	1.174*	(0.0873)	1.161	(0.119)	1.231*	(0.130)
Children						
None	REF	REF	REF	REF	REF	REF
1-2	1.035	(0.0929)	0.873	(0.108)	1.164	(0.125)
3 or more	0.829*	(0.0665)	0.872	(0.103)	0.791*	(0.0846)
Past week employment						
Yes	1.342**	(0.133)	0.856	(0.144)	1.323*	(0.178)
None, looking for work	REF	REF	REF	REF	REF	REF
None, not looking for work	1.488***	(0.132)	0.821	(0.137)	1.567***	(0.163)
**Acculturation**						
Years in the US						
5 or fewer	REF	REF	-	-	-	-
6 or more	1.348***	(0.0985)	-	-	-	-
English at home						
No	REF	REF	REF	REF	REF	REF
Yes	1.269**	(0.0971)	0.684**	(0.0802)	1.590***	(0.173)
Observations	492		244		239	

*p < 0.05, **p < 0.01, ***p < 0.001.

aIRR = adjusted incidence rate ratio.

In terms of measures of acculturation, respondents who have been living in the US for six years or more reported higher rates of adverse effects in their communities than counterparts here for less time (aIRR = 1.35, p < 0.001) (Table 3). Individuals who spoke English at home had 1.27 times higher rates of adverse community effects than those who did not speak English at home (p < 0.01).

Stratification of the primary outcome of interest model by years in the US showed that all effects significant in the overall model were of comparable size and significance for the sample of respondents who had been in the US for at least six years. Among respondents who were newer immigrants, the only factor associated with increased reporting of adverse community effects was speaking English at home, which was associated with lower rates of adverse community effects (aIRR = 0.68, p < 0.01) (Table 3).

#### 3.2.2 Individual adverse events

Multivariable results for our secondary outcomes of interest, individual adverse community effects, indicate that younger respondents reported more adverse outcomes in every community effect assessed with the exception of household violence (aORs of 0.97 for all) (Table 4). Females were much more likely to report increases in food insecurity and school interruptions in their communities (aOR = 2.44, p < 0.01 and aOR = 4.42, p < 0.001, respectively). Males, however, were more than three times more likely to report increased job/wage loss in their communities than females (female aOR = 0.26, p < 0.001). The number of children respondents had was not associated with individual outcomes with the exception of increased community food insecurity and increased community substance misuse, both of which were reported with less frequency among respondents with three or more children, relative to those with no children (aOR = 0.42, p < 0.01 and aOR = 0.40, p < 0.05, respectively). Relative to being unemployed but seeking work, respondents who were employed or who were not employed and not seeking work were both more likely to report community increases in job or wage loss (employed only, aOR = 2.55, p < 0.05), bank or cash barriers (employed aOR = 2.96, p < 0.05; unemployed and not looking for work aOR = 3.64, p < 0.001), school interruptions (employed aOR = 3.81, p < 0.001); unemployed and not looking for work aOR = 3.63, p < 0.001), substance misuse (only unemployed and not looking for work, aOR = 3.57, p < 0.01) and poor mental health (only unemployed and not looking for work, aOR = 2.86, p < 0.001).

**Table 4:** Multivariable logistic regression models exploring factors associated with individual adverse community effects experienced during the COVID-19 pandemic by refugee communities in San Diego, California.

	Job/Wage loss		Bank/cash barriers		Food insecurity		School interruptions		Household violence		Substance misuse		Poor mental health	
	aOR	SE	aOR	SE	aOR	SE	aOR	SE	aOR	SE	aOR	SE	aOR	SE
**Sociodemographics**														
Age (years)	0.971**	(0.0107)	0.965***	(0.0104)	0.973*	(0.0120)	0.968**	(0.0101)	0.979	(0.0134)	0.965***	(0.0102)	0.974**	(0.00830)
Gender														
Male	REF	REF	REF	REF	REF	REF	REF	REF	REF	REF	REF	REF	REF	REF
Female	0.256***	(0.0908)	1.746	(0.551)	2.440**	(0.784)	4.415***	(1.309)	1.952	(0.834)	1.342	(0.425)	1.273	(0.275)
Children														
None	REF	REF	REF	REF	REF	REF	REF	REF	REF	REF	REF	REF	REF	REF
1-2	1.535	(0.561)	1.045	(0.353)	0.760	(0.258)	1.304	(0.404)	1.185	(0.512)	1.157	(0.417)	1.004	(0.262)
3 or more	0.758	(0.265)	0.592	(0.203)	0.418**	(0.141)	1.066	(0.353)	0.375	(0.228)	0.401*	(0.156)	0.668	(0.166)
Past week employment														
Yes	2.552*	(1.044)	2.963*	(1.288)	0.796	(0.300)	3.808***	(1.518)	0.944	(0.570)	2.353	(1.156)	1.682	(0.476)
None, looking for work	REF	REF	REF	REF	REF	REF	REF	REF	REF	REF	REF	REF	REF	REF
None, not looking for work	1.640	(0.500)	3.637***	(1.401)	1.083	(0.346)	3.627***	(1.339)	1.749	(0.828)	3.566**	(1.515)	2.859***	(0.742)
**Acculturation**														
Years in the US														
5 or fewer	REF	REF	REF	REF	REF	REF	REF	REF	REF	REF	REF	REF	REF	REF
6 or more	1.068	(0.370)	2.632**	(0.811)	3.793***	(1.387)	1.886*	(0.573)	4.516**	(2.562)	2.741**	(0.947)	1.579	(0.371)
English at home														
No	REF	REF	REF	REF	REF	REF	REF	REF	REF	REF	REF	REF	REF	REF
Yes	0.637	(0.218)	2.038*	(0.617)	2.635**	(0.784)	2.827***	(0.764)	8.714***	(4.975)	2.544**	(0.856)	0.872	(0.199)
Observations	483		483		483		483		483		483		483	

*p < 0.05, **p < 0.01, ***p < 0.001.

aOR = adjusted odds ratio.

Respondents who had lived in the US for 6 years or more were more likely to report that every assessed community effect had gotten worse with the exceptions of job/wage loss and mental health (bank/cash barriers aOR = 2.63, p < 0.01; food insecurity aOR = 3.79, p < 0.001; school interruptions aOR = 1.89, p < 0.01; household violence aOR = 4.52, p < 0.01; substance misuse aOR = 2.74, p < 0.01) (Table 4). Respondents who spoke English at home were more likely to report increased in bank/cash barriers (aOR = 2.04, p < 0.05), food insecurity (aOR = 2.64, p < 0.01), school interruptions (aOR = 2.83, p < 0.001), household violence (aOR = 8.71, p < 0.001) and substance misuse (aOR = 2.54, p < 0.01).

#### 3.2.3 Individual adverse events stratified by duration of residence in US

Stratification of the secondary outcomes of interest by duration of residence in the US showed relatively few significant associations among newer immigrants. Job and/or wage loss were less likely to be reported by newer immigrants who were older (aOR = 0.94, p < 0.05) (Table 5). Females residing in the US for five or fewer years were less likely to report increases in community job or wage loss (aOR = 0.12, p < 0.001), and more likely to report increases in community food insecurity (aOR = 14.95, p < 0.001) and school interruptions (aOR = 10.38, p < 0.001). Newer immigrants who spoke English at home were less likely to report increases in job or wage loss (aOR = 0.23, p < 0.05), substance misuse (aOR = 0.11, p < 0.05) or poor mental health (aOR = 0.36, p < 0.01).

**Table 5:** Multivariable logistic regression models exploring factors associated with individual adverse community effects experienced during the COVID-19 pandemic by refugee communities in San Diego, California who have lived in the US for five or fewer years.

	Job/Wage loss		Bank/cash barriers		Food insecurity		School interruptions		Household violence		Substance misuse		Poor mental health	
	aOR	SE	aOR	SE	aOR	SE	aOR	SE	aOR	SE	aOR	SE	aOR	SE
**Sociodemographics**														
Age (years)	0.936*	(0.0252)	0.963	(0.0244)	1.016	(0.0288)	0.989	(0.0231)	0.987	(0.0206)	0.955	(0.0264)	1.009	(0.0146)
Gender														
Male	REF	REF	REF	REF	REF	REF	REF	REF	REF	REF	REF	REF	REF	REF
Female	0.123***	(0.0593)	1.590	(1.078)	14.95***	(9.865)	10.38***	(4.443)	0.615	(0.907)	0.182	(0.209)	1.017	(0.338)
Children														
None	REF	REF	REF	REF	REF	REF	REF	REF	REF	REF	REF	REF	REF	REF
1-2	0.779	(0.707)	1.329	(1.140)	0.0593**	(0.0638)	0.797	(0.551)	1.028	(1.331)	0.240	(0.260)	0.826	(0.369)
3 or more	0.571	(0.509)	1.662	(1.407)	0.627	(0.401)	0.679	(0.437)	1.189	(1.403)	0.592	(0.443)	0.597	(0.242)
Past week employment														
Yes	0.910	(0.770)	1.493	(1.916)	0.235	(0.243)	1.882	(1.580)	1	(.)	0.329	(0.326)	0.840	(0.422)
None, looking for work	REF	REF	REF	REF	REF	REF	REF	REF	REF	REF	REF	REF	REF	REF
None, not looking for work	0.395	(0.305)	1.257	(1.482)	0.159	(0.149)	1.404	(1.147)	0.0482*	(0.0705)	0.297	(0.265)	0.927	(0.457)
**Acculturation**														
English at home														
No	REF	REF	REF	REF	REF	REF	REF	REF	REF	REF	REF	REF	REF	REF
Yes	0.233*	(0.137)	0.147	(0.176)	0.373	(0.367)	0.772	(0.447)	0.608	(0.726)	0.106*	(0.115)	0.357**	(0.141)
Observations	244		244		244		244		169		244		244	

*p < 0.05, **p < 0.01, ***p < 0.001.

aOR = adjusted odds ratio.

Residents who have been in the US for at least six years had more consistent associations across individual assessed community adverse effects. Older respondents were less likely to report bank/cash barriers (aOR = 0.97, p < 0.05), food insecurity (aOR = 0.97, p < 0.05), school interruptions (aOR = 0.97, p < 0.01), substance misuse (aOR = 0.96, p < 0.01) and poor mental health (aOR = 0.96, p < 0.001) (Table 6). Females were less likely to report increases in job/wage loss (aOR = 0.38, p < 0.05) and more likely to report school interruptions (aOR = 2.71, p < 0.01) or substance misuse (aOR = 2.67, p < 0.05). Respondents who have been in the US for six or more years and had three or more children were less likely to report bank cash barriers (aOR = 0.33, p < 0.05), food insecurity (aOR = 0.29, p < 0.01) and substance misuse (aOR = 0.09, p < 0.05). Respondents who were unemployed and not looking for work were more likely to report community job/wage loss (aOR = 2.14, p < 0.01), bank/cash barriers (aOR = 3.42, p < 0.01), school interruptions (aOR = 4.29, p < 0.001), substance misuse (aoR = 4.81, p < 0.01) and poor mental health (aOR = 4.47, p < 0.001) than respondents who were unemployed and looking for work. Current employment was also associated with higher likelihood of reporting community increases in school interruptions (aOR = 3.87, p < 0.01). Respondents who had lived in the US for at least 6 years and spoke English at home were more likely to report community bank/cash barriers (aOR = 4.19, p < 0.01, food insecurity (aOR = 3.39, p < 0.001), school interruptions (aOR = 4.93, p < 0.001), household violence (aOR = 10.72, p < 0.001) and substance misuse (aOR = 5.67, p < 0.001).

**Table 6:** Multivariable logistic regression models exploring factors associated with individual adverse community effects experienced during the COVID-19 pandemic by refugee communities in San Diego, California who have lived in the US for six or more years.

	Job/Wage loss		Bank/cash barriers		Food insecurity		School interruptions		Household violence		Substance misuse		Poor mental health	
	aOR	SE	aOR	SE	aOR	SE	aOR	SE	aOR	SE	aOR	SE	aOR	SE
**Sociodemographics**														
Age (years)	0.977	(0.0129)	0.967*	(0.0134)	0.969*	(0.0139)	0.966**	(0.0123)	0.976	(0.0152)	0.963**	(0.0129)	0.955***	(0.0119)
Gender														
Male	REF	REF	REF	REF	REF	REF	REF	REF	REF	REF	REF	REF	REF	REF
Female	0.377*	(0.167)	2.244	(0.934)	1.598	(0.587)	2.712**	(1.046)	2.317	(1.162)	2.669*	(1.294)	1.784	(0.591)
Children														
None	REF	REF	REF	REF	REF	REF	REF	REF	REF	REF	REF	REF	REF	REF
1-2	2.519	(1.239)	1.182	(0.485)	1.113	(0.426)	1.352	(0.509)	1.311	(0.611)	1.975	(0.836)	1.139	(0.410)
3 or more	0.784	(0.327)	0.326*	(0.180)	0.290**	(0.130)	1.551	(0.648)	0.265	(0.217)	0.0933*	(0.0977)	0.763	(0.265)
Past week employment														
Yes	2.930	(1.667)	2.586	(1.461)	0.834	(0.386)	3.867**	(1.882)	1.589	(1.111)	2.398	(1.523)	1.450	(0.572)
None, looking for work	REF	REF	REF	REF	REF	REF	REF	REF	REF	REF	REF	REF	REF	REF
None, not looking for work	2.144*	(0.825)	3.420**	(1.613)	1.267	(0.480)	4.286***	(1.849)	2.829	(1.534)	4.811**	(2.421)	4.465***	(1.696)
**Acculturation**														
English at home														
No	REF	REF	REF	REF	REF	REF	REF	REF	REF	REF	REF	REF	REF	REF
Yes	0.772	(0.341)	4.185**	(1.837)	3.385***	(1.250)	4.933***	(1.846)	10.72***	(7.560)	5.671***	(2.890)	1.437	(0.487)
Observations	239		239		239		239		239		239		239	

*p < 0.05, **p < 0.01, ***p < 0.001.

aOR = adjusted odds ratio.

#### 3.2.4 Number of adverse events stratified by country of birth

Exploratory regressions stratified by country of birth among the three countries with the largest number of respondents (Syria, Somalia, and Afghanistan). In these country-specific regressions, age, gender and the number of children were not significantly related to number of adverse community effects (Table 7). Among respondents born in Somalia, relative to people who were unemployed but looking for work, those who were unemployed reported increases in the incidence rate of adverse community effects (aIRR = 1.69, p < 0.001); respondents born in Syria who were employed or were unemployed and not looking for work reported decreases in the rate of adverse community effects (aIRR = 0.71, p < 0.05 and aIRR = 0.68, p < 0.05, respectively). Longer durations of time since immigration to the US was associated with increases in reporting higher incidences of adverse community effects for respondents born in Syria (aIRR = 1.29, p < 0.01 for six or more years vs. five or fewer years); there was no significant association for respondents born in Somalia or Afghanistan. Speaking English at home was associated with much higher increases in rates of adverse community effects reported by respondents born in Somalia (aIRR = 3.60, p < 0.001).

**Table 7:** Multivariable Poisson regression models exploring factors associated with the number of adverse community effects experienced during the COVID-19 pandemic by refugee communities in San Diego, California, stratified by the three most prevalent countries of birth.

	Country of birth					
	Syria		Somalia		Afghanistan	
	IRR	SE	IRR	SE	IRR	SE
**Sociodemographics**						
Age (years)	0.995	(0.00404)	0.996	(0.00542)	1.006	(0.00436)
Gender						
Male	REF	REF	REF	REF	REF	REF
Female	0.957	(0.136)	1.081	(0.154)	1.128	(0.203)
Children						
None	REF	REF	REF	REF	REF	REF
1-2	0.995	(0.137)	1.247	(0.181)	0.806	(0.112)
3 or more	0.939	(0.104)	1.082	(0.185)	0.947	(0.132)
Past week employment						
Yes	0.705*	(0.120)	1.057	(0.265)	0.896	(0.140)
None, looking for work	REF	REF	REF	REF	REF	REF
None, not looking for work	0.648*	(0.113)	1.692***	(0.246)	0.813	(0.125)
**Acculturation**						
Years in the US						
5 or fewer	REF	REF	REF	REF	REF	REF
6 or more	1.293**	(0.123)	0.867	(0.225)	0.866	(0.106)
English at home						
No	REF	REF	REF	REF	REF	REF
Yes	0.969	(0.144)	3.604***	(0.487)	0.772	(0.110)
Observations	150		110		80	

*p < 0.05, **p < 0.01, ***p < 0.001.

## 4 Discussion

Overall, this sample of refugee community members from San Diego, California reported substantial, adverse COVID-19 related effects, with increases in levels of job or wage loss and poor mental health being most common. Adverse community effects were concentrated in respondents living in the US for six or more years, who had a more than 150% higher number of average adverse community effects of COVID-19 than newer immigrants.

The distinction of five years or fewer vs. six or more in the US may be driven by several primary factors. First, refugees receive modest governmental and NGO support when they first arrive in the US, comprising a period of more intensive support for 90 days and case management for up to eight months (Office of Refugee Resettlement, 2022b). Additionally, for up to five years, refugees are able to receive services via Office of Refugee Resettlement grants to nonprofits that generally focus on job placement and language skills (Office of Refugee Resettlement, 2022a; 2022b). This support may help to temper the adverse community effects experienced during the COVID-19 pandemic for newer immigrants, but ends abruptly at the five year mark, leaving those individuals who have not been able to create economic and social inroads and connections without adequate support. The lack of additional support likely further exacerbated the effects of the COVID-19 pandemic on individuals who have been in the US for longer periods of time, for example leaving those who lost service sector jobs during the pandemic with no benefits on which to fall back.

Second, as refugees are in the United States for longer periods of time, not only are they eligible for fewer sources of additional support, but they are increasingly acculturated, and may be more aware of, or open to discussing, issues such as household violence and substance misuse than new immigrants (Dalgaard & Montgomery, 2015; van Os et al., 2020). In tandem, refugees in PANA-served communities have discussed the emotional process associated with resettlement, characterized by an initial gratitude for having survived the turmoil and strife in their country of origin and being given a second chance, followed by a waning of that gratitude as the realities of struggles in their new home set in (Healey, 2014; McMichael et al., 2017). These two explanations are supported in part by the fact that both community food insecurity and substance misuse use were substantially lower in San Diego refugee community respondents who had been in the US for five years or fewer.

Job losses among refugee respondents and in their communities were substantially and adversely impacted by the COVID-19 pandemic. Levels of increased job or wage loss in the community during the pandemic were nearly 30 percentage points higher in the San Diego refugee community than were seen statewide or in the southern California region. Survey respondents who were not seeking to change their employment status (i.e., currently employed or currently unemployed and not looking for work) reported more adverse effects of COVID-19 in their communities, compared with counterparts actively seeking to change their employment status (i.e., unemployed but looking for work). Unemployed respondents actively seeking work were overwhelmingly those who had been in the US for six or more years (71%, vs. 29% among more recent immigrants). Additional community conversations regarding contextual factors surrounding employment status, desires and opportunities are needed to better understand these distinctions.

Refugees often live on relatively limited incomes, and are typically employed in more marginal jobs (i.e. those with lower wages, lower job security and lower benefits, such as taxi and rideshare drivers, restaurant workers, and childcare providers) that have been particularly vulnerable to the economic oscillations seen over the course of the COVID-19 pandemic. They may therefore be at greater risk of job loss, and of having more adverse financial consequences if jobs are lost, making unemployed respondents looking for work perhaps more focused on their employment search rather than community observation and connection. This economic vulnerability is in large part due to a policy drive for refugees to become self-sufficient through early employment (Brown & Scribner, 2014), compromising their abilities to gain the skills needed to get a job that would provide a more robust and reliable income for their families.

Overall, nearly half of all survey respondents (49%) indiated that mental health in their communities had worsened during COVID-19, a similar prevalence to that seen in Californians overall (52%). However, in our sample, these levels were divergent based on years in the US, with respondents who had been in the US for five or fewer years reporting lower levels of COVID-19 related adverse mental health in the community than those here for six or more years (44% vs. 54%). People who were unemployed and not looking for work, and younger respondents, both reported worse levels of mental health in their communities; these associations were significant only among respondents who have lived in the US for six or more years. These findings are in line with the mental shifts associated with long-term resettlement, in which many refugees transition from a perspective of hope at being accepted into a new country and leaving a place of persecution, political instability, disaster or war, into a perspective of hopelessness at dealing with the trauma they experienced in their countries of origin and living in a country where economic insecurity, cultural stressors, and social and structural discrimination present ongoing barriers (Healey, 2014; Marshall et al., 2005; McMichael et al., 2017; Uribe Guajardo et al., 2016). It has also been suggested that in addition to serving as a proxy for acculturation, longer-term residence in a host country may be a marker for the manifestation of protracted discrimination and isolation which additionally compound adverse mental health (Gleeson et al., 2020).

These findings have direct implications for policy and practice aimed to improve refugee health in the US. There is a clear need for well-structured, cohesive support mechanisms for refugee communities that offer upstream structural shifts to prevent downstream adverse effects. These shifts are all the more urgent given the Biden Administrations’ commitment to admit greater numbers of refugees (The White House, 2022). The support included in the US Refugee Act has been substantially curtailed since its enactment in 1980, and has historically included a strong push towards early employment (Bruno, 2011; United States Congress, 1980). While employment is an integral step in supporting independence and self-sufficiency, early employment in the absence of strategic training and placement increases the likelihood that refugees find low-wage employment with limited job security and benefits, and has consequently made these communities more vulnerable to the employment losses experienced during the COVID-19 pandemic. Policies to support and facilitate the identification of available, higher paying jobs, and the professional upskilling required to obtain those jobs (some of which may not require a college degree), will offer refugees and their families substantially more security and stability, and increase their opportunities to be independent and to thrive in this country. Additionally, employment initiatives in the absence of other needs such as food security, education, housing, and physical and mental health care, may undermine the holistic foundation needed to build and sustain a new life. Refugee community organizations who offer these single-topic programs should explore options to either expand their programs into more comprehensive offerings, or meaningfully and functionally partner with other organizations whose programs are able to complement their own offerings.

Younger respondents reported higher levels of adverse community effects overall, and in seven of eight individual effects assessed. Given that the mean age of respondents in this sample was 40, but ranged from 18 to 96, younger respondents may be more aware of changes brought on in their communities by the COVID-19 pandemic because the adverse effects assessed focus on income generation and school, factors more likely to affect younger respondents (Kaugars et al., 2022). Additionally, older respondents may have experienced more severe trauma from factors such as war, persecution and natural disasters in their home countries, potentially making them less sensitive to issues such as COVID. We have also been told by our community partners that when they do watch the news, it is more often news in their home countries. Younger respondents, in contrast, may be more involved in their communities to notice the broader effects of COVID, and may also be more fearful of COVID, in the absence of the traumatic experiences of older refugee community members.

Female respondents in this sample reported more adverse community effects of COVID-19 than men, and those responses were significant for seeing more food insecurity and school interruptions. This is very much in line with findings from a wide range of settings which find that women's burden of unpaid care has escalated dramatically during this pandemic (Chauhan, 2020; Seedat & Rondon, 2021; Xue & McMunn, 2021), and that food insecurity is of increasing concern to women trying to feed their families in the context of increasing food prices and mobility restrictions (Belsey-Priebe et al., 2021; Hamadani et al., 2020). While the questions in this survey were asking respondents about what they noticed in their communities, rather than their personal experiences, women may be more aware of issues and concerns facing other women in their communities who are similar to themselves (the concept of social network homophily) (McPherson et al., 2001).

These gender differences may also reflect cultural differences based on countries of birth (Dryden-Peterson, 2016), as the majority of respondents from Somalia and Myanmar were female, while the majority of respondents from Syria and Afghanistan were men. We opted to not adjust for country of birth in our regression analyses, as the diversity of countries represented would have required an aggregation of origin countries that obscured important cultural and contextual differences. Our country of birth-stratified analyses for Syria, Somalia and Afghanistan indicate no significant associations between gender and the overall number of adverse community events reported, and sample sizes do not allow for adverse community event-specific stratified regressions. This distinction merits further research with larger sample sizes.

These findings should be interpreted in light of certain limitations. This sample was drawn from community members who had engaged with PANA in the previous year and had time to respond to survey questions, and is not representative of the entire refugee community in San Diego, nor of refugee communities elsewhere. Additionally, respondents were from a wide range of countries, and our sample was not large enough to analyze these data independently for each of those countries of origin. From a cultural perspective, refugee communities represented in these data may also be more hesitant to discuss issues that are considered taboo or undesirable, such as substance misuse and domestic violence. All data are self-report and therefore subject to desirability bias. Finally, acculturation could only be measured by proxy; longer acculturation scales were not included in this survey instrument due to length limitations.

This study highlights the substantial impact that the COVID-19 pandemic is having on refugee communities in San Diego. Refugee community respondents note much higher levels of job and/or wage loss than seen in the state of California at large. Recent immigration appears to be a buffer for many of the adverse community effects of COVID-19, though further research is needed to determine if that is due to supportive factors (e.g. community support organizations, governmental support, resettlement assistance), comparative factors (e.g. comparison to the circumstances that necessitated immigration) or other reasons. Members of the refugee community who have been in the US for longer periods of time appear particularly vulnerable to the negative impacts of this pandemic. Policy facilitating the creation of cohesive, upstream, holistic refugee support structures is needed to better equip refugees for success and independence in their new homes, so that they do not flounder when they are no longer eligible for support through existing mechanisms. These structures are particularly key in the context of the COVID-19 pandemic, as refugees with different levels of acculturation grapple with the repercussions of this societal shock wave that is likely to be with us for many years to come.

## Author statement

**Lotus McDougal**: Conceptualization, Methodology, Formal Analysis, Writing – Original Draft, Writing – Review & Editing, Visualization **Jeanine Erikat**: Investigation, Resources, Writing – Review & Editing, Project administration **Homayra Yusufi**: Conceptualization, Investigation, Resources, Writing – Review & Editing, Project administration, Funding acquisition **Ramla Sahid**: Conceptualization, Investigation, Resources, Writing – Review & Editing, Project administration, Funding acquisition **Samantha Streuli**: Writing – Review & Editing **Rebecca Fielding-Miller**: Conceptualization, Investigation, Data Curation, Writing – Review & Editing, Supervision, Project administration, Funding acquisition.

## Funding

Funding for this research was provided by the California Endowment and Satterberg Foundation. Additional support for analyses was provided by a UCSD Dissemination and Implementation Science Core pilot grant and the 10.13039/100000002National Institutes of Health (K01MH112436, U01HD108787).

## Ethics

These data were collected as part of the Partnership for the Advancement of New American's (PANA) biannual Refugee Experiences Report. The study was reviewed and approved by the University of California, San Diego Institutional Review Board. All elements of study and survey design were led by PANA staff (RS, JE, HY) with technical support from RFM. Participants provided informed consent before completing the survey and at completion were given a $15 gift card to thank them for their time and expertise.

## Declaration of competing interest

None.
